# Knowledge, Attitudes, and Stigma Towards People Living with HIV: An Explanatory Sequential Mixed-Methods Study Among 1013 Healthcare Professionals in Spain

**DOI:** 10.3390/healthcare14060737

**Published:** 2026-03-13

**Authors:** Yelson Alejandro Picón-Jaimes, Ivan David Lozada-Martínez, Sulaiman Kalokoh, Mar Rosàs Tosas, Juan Tiraboschi

**Affiliations:** 1 Blanquerna School of Health Sciences, Ramon Llull University, 08022 Barcelona, Spain; mariadelmarrt1@blanquerna.url.edu; 2 Biomedical Scientometrics and Evidence-Based Research Unit, Department of Health Sciences, Universidad de la Costa, Barranquilla 080002, Colombia; ivandavidloma@gmail.com; 3 Clínica Colsanitas S.A., Clínica Iberoamérica, Barranquilla 080001, Colombia; 4 Faculty of Information and Communication Technology, Limkokwing University of Creative Technology, Freetown 00232, Sierra Leone; kalokohsulaiman@gmail.com; 5 Infectious Diseases Service, Bellvitge-IDIBELL-University Hospital, University of Barcelona, 08870 Hospitalet de Llobregat, Spain; jmtiraboschi@bellvitgehospital.cat

**Keywords:** social stigma, prejudice, HIV, acquired immunodeficiency syndrome

## Abstract

**Highlights:**

**What are the main findings?**

- Healthcare professionals in Spain show high factual knowledge about HIV transmission and treatment, yet more than half report insufficient training and feel unprepared to care for people living with HIV.
- Despite generally positive attitudes, fear of contagion and subtle stigmatizing behaviors persist, driven mainly by lack of training and cultural prejudices.

**What are the implications of the main findings?**

- Adequate knowledge alone is insufficient to eliminate HIV-related stigma in healthcare settings; structured and continuous training is essential to translate knowledge into stigma-free practice.
- Targeted educational interventions, interprofessional training, and institutional commitment are key to improving the quality, equity, and humanization of care for people living with HIV.

**Abstract:**

**Background/Objectives:** Stigma and fear related to human immunodeficiency virus persist in healthcare settings and negatively influence professionals’ attitudes and the quality of care provided to people living with human immunodeficiency virus. This study aimed to evaluate knowledge, attitudes, and stigma toward people living with human immunodeficiency virus among healthcare professionals in Spain and to explore strategies to reduce stigma. **Methods:** An explanatory sequential mixed-methods study was conducted. In the quantitative phase, an online questionnaire based on the International Planned Parenthood Federation instrument was disseminated nationwide through social media using non-probability convenience sampling. Quantitative data from 1013 healthcare professionals were analyzed using descriptive statistics and non-parametric tests (Kruskal–Wallis, chi-square, Friedman) with appropriate corrections for multiple comparisons. In the qualitative phase, 12 participants were purposively selected for semi-structured interviews to explain quantitative findings. Qualitative data were analyzed using reflexive thematic analysis. Integration occurred through joint interpretation and a joint display table connecting quantitative patterns with qualitative themes. Ethical approval was obtained from the Clinical Research Ethics Committee of Bellvitge Hospital in Catalonia. **Results**: A total of 1013 healthcare professionals from diverse specialties participated, and twelve completed qualitative interviews. Knowledge regarding transmission, prevention, and treatment of human immunodeficiency virus was high. However, more than half reported no specific training and felt unprepared to care for people living with human immunodeficiency virus. Despite knowledge, fear of contagion was common. Attitudes were positive, with acceptance of caring for people living with human immunodeficiency virus and rejection of common misconceptions. Qualitative findings revealed persistent stigma linked to insufficient training and cultural prejudice. Integration of quantitative and qualitative data revealed that knowledge alone does not eliminate fear, and that the gap between theoretical understanding and clinical confidence represents a critical barrier to stigma-free care. **Conclusions:** Although healthcare professionals in Spain demonstrate knowledge about human immunodeficiency virus, stigma and fear remain prevalent. Targeted education and interprofessional training are needed to ensure respectful, inclusive, and stigma-free clinical care.

## 1. Introduction

Human immunodeficiency virus (HIV) infection has been stigmatized since its discovery, beginning with the erroneous label of “gay syndrome,” which contributed to discrimination against a specific group of the population [1,2,3]. In this study, stigma is operationally defined following Link and Phelan’s conceptualization as a social process involving labeling, stereotyping, separation, status loss, and discrimination, manifested in healthcare settings through avoidance behaviors, differential treatment, breaches of confidentiality, and negative attitudes that compromise the quality and equity of care provided to PLWH [4,5]. People living with HIV (PLWH) face this type of segregation, especially when seeking healthcare [6,7].

Previous studies, for example, one from 2016, which evaluated 713 health workers, revealed that as age increased, acceptance of patients with HIV/AIDS decreased, and fear increased. Physicians and surgical department staff showed significantly higher levels of discrimination and fear towards these patients [8]. Another Spanish study from 2020 evaluated the attitudes of 284 nursing students towards people living with HIV, finding that the majority had positive attitudes [9]. This reinforces the idea that the humanistic approach to nursing contributes to generating a favorable perception towards this population [9]. A European multicenter study with 594 nursing students revealed that despite knowing how the virus is transmitted, about 50% of the participants feared becoming infected [10]. In addition, about 15% of the participants felt angry about the risk that men who have sex with men represent to the heterosexual community, and a significant percentage had difficulties being understanding towards HIV patients [10].

Gaps in understanding stigma and prejudice persist in the Spanish context. This stigmatization can have serious repercussions, decreasing the willingness of PLWH to seek care and negatively affecting public health, as those who fear being stigmatized are less likely to treat their condition appropriately [11,12,13].

This study therefore aimed to analyze the knowledge, attitudes, and stigma towards people living with HIV in the Spanish healthcare setting and, through a mixed-methods approach, to identify opportunities for improving understanding and reducing stigma.

## 2. Materials and Methods

### 2.1. Study Design

A mixed-methods study was conducted, comprising a quantitative cross-sectional phase and a qualitative phase with semi-structured interviews following a phenomenological approach. The Strengthening the Reporting of Observational Studies in Epidemiology (STROBE) guidelines were applied to the quantitative cross-sectional phase, as they are recommended for such study designs, while the Consolidated Criteria for Reporting Qualitative Research (COREQ) guidelines were applied to the qualitative phase.

Thus, a descriptive research method was employed in the quantitative phase to assess proportions and characterize the population studied.

The justification for using a mixed-methods design was to obtain complementary perspectives: the quantitative phase allowed the measurement of knowledge, attitudes, and stigma through standardized indicators, while the qualitative phase provided an in-depth understanding of the lived experiences, contextual factors, and meanings attached to stigma in practice. This study employed an explanatory sequential mixed-methods design, in which quantitative data were collected and analyzed first, followed by qualitative interviews designed to explain and contextualize the quantitative findings. Integration occurred at the interpretation stage, where qualitative themes were used to explain patterns observed in the quantitative data, particularly regarding the persistence of fear despite high knowledge levels and the gap between training and preparedness.

### 2.2. Setting

The study was conducted in the Spanish healthcare setting, covering public and private hospitals and medical centers. It focused on healthcare professionals who interacted with PLWH.

### 2.3. Participants

In the quantitative phase, the inclusion criteria were healthcare professionals over 18 years of age who cared for PLWH, consented to participate, and were authorized to practice in Spain. The exclusion criteria were retired professionals and those engaged solely in administrative work. The final sample comprised 1013 professionals, exceeding the minimum required sample size. A non-probability convenience sampling method was used in the quantitative phase, recruiting participants through an open invitation disseminated via social media to maximize geographic coverage. This approach allowed for a broad geographic reach but does not permit statistical generalization to the entire population of healthcare professionals in Spain.

In the qualitative phase, the inclusion criteria were healthcare professionals who had participated in the quantitative phase and provided their consent to be contacted for further interviews. Purposive sampling was used to select participants who had completed the quantitative survey and indicated willingness to participate in an interview. Selection aimed to achieve variation in profession (physicians, nurses, technicians, psychologists, allied health), years of experience (range: 3–30 years), gender, and geographic region. Twelve professionals were contacted based on these criteria, and all accepted participation. Thematic saturation was assessed iteratively during data collection; after the tenth interview, no substantially new themes emerged, and two additional interviews were conducted to confirm saturation.

### 2.4. Variables

Quantitative variables were derived from the International Planned Parenthood Federation’s “Attitudes and Practices for Health Care Providers and Other Personnel Regarding HIV/AIDS” (CAP HIV/AIDS) questionnaire. These variables included demographic aspects and assessed professional experience, knowledge about HIV, training received, attitudes towards associated stigma, and fear of contagion. In this context, knowledge, attitudes, and perceptions of stigma were considered the dependent variables, while sociodemographic and professional characteristics (e.g., age, gender, years of experience, type of profession, and prior training) were treated as independent variables. Qualitative variables included personal definition of stigma, experiences of stigma, impact on the doctor-patient relationship, factors contributing to stigma, and strategies to address it and improve care.

### 2.5. Data Sources/Measurement

The quantitative questionnaire contained closed questions and Likert-type scales. Data were collected through an online self-administered questionnaire distributed via Google Forms^®^ (Google LLC, Mountain View, CA, USA), which allowed wide dissemination across Spain while ensuring secure and anonymous responses. Data collection was carried out anonymously to ensure confidentiality.

The quantitative instrument was based on the International Planned Parenthood Federation’s “Attitudes and Practices for Health Care Providers and Other Personnel Regarding HIV/AIDS” (CAP HIV/AIDS) questionnaire. This instrument has been used internationally to assess knowledge, attitudes, and practices related to HIV among healthcare workers.

For the Spanish context, the following adaptation process was undertaken: (1) forward translation from English to Spanish by two independent bilingual health professionals; (2) reconciliation of translations and cultural adaptation of items to ensure appropriateness for the Spanish healthcare context; (3) back-translation to English by an independent translator; (4) expert review by three HIV specialists and two qualitative methodology experts to assess content validity and cultural relevance; and (5) pilot testing with 20 healthcare professionals from diverse specialties to assess comprehension, acceptability, and completion time.

Based on pilot feedback, minor wording adjustments were made to improve clarity. Given the descriptive nature of the study and the use of single items to assess specific knowledge points and attitudes, formal psychometric validation (e.g., factor analysis) was not conducted. However, internal consistency was assessed for conceptually related item groups:

- Knowledge items (transmission routes, prevention methods, treatment): Cronbach’s α = 0.78
- Attitude items (rights of PLWH, willingness to provide care): Cronbach’s α = 0.81
- Fear/stigma items (concern about contagion, rejection, judgment): Cronbach’s α = 0.74

These values indicate acceptable internal consistency for exploratory research purposes. Results are presented primarily at the item level to provide specific, actionable insights for training and intervention development, while also being grouped into conceptual domains (knowledge, attitudes, fear/stigma) for interpretive purposes.

Response options for knowledge items were “Yes”, “No”, and “Not sure”. Attitude and fear items used a 4-point Likert scale: “Not at all”, “A little”, “A little more than usual”, and “A lot”. The “Not sure” option for knowledge items was retained in the analysis as it provides meaningful information about uncertainty, which may indicate training gaps.

In the qualitative phase, semi-structured interviews of approximately 60 min were conducted via video call between [November 2024 and May 2025]. The interview guide was developed by the research team based on quantitative findings and literature review, covering the following domains: personal definition of stigma, experiences witnessing or enacting stigma, impact on patient-provider relationships, perceived barriers to stigma-free care, and strategies for improvement.

The interview guide was reviewed by two experts in qualitative methodology and piloted with three healthcare professionals not included in the final sample. Minor modifications were made to improve question flow and clarity.

All interviews were conducted by the first author (YAPJ), who is a physician with training in qualitative interviewing methods. The interviewer had no prior clinical or professional relationship with participants. At the beginning of each interview, the interviewer’s role and study objectives were explained, and participants were assured of confidentiality and their right to withdraw.

Interviews were audio-recorded with explicit participant consent and transcribed verbatim by a professional transcription service. Transcripts were anonymized and returned to participants for member checking; participants were invited to clarify, expand, or retract any statements. Three participants provided minor clarifications, which were incorporated into the final transcripts.

### 2.6. Bias

Several potential sources of bias must be acknowledged in this study. Coverage bias is a significant concern, as recruitment through social media platforms may have systematically excluded healthcare professionals with limited digital engagement, older professionals less active on social media, or those working in settings with restricted internet access. This may have resulted in a sample skewed toward younger, more digitally connected professionals.

Self-selection bias is inherent to the voluntary nature of participation. Professionals with a greater interest in HIV care, prior experience with PLWH, or stronger opinions about stigma may have been more likely to participate, potentially leading to overestimation of knowledge and underestimation of stigma compared to the general population of healthcare professionals.

Social desirability bias is particularly relevant for stigma-related items. Despite anonymity, participants may have provided responses they perceived as socially or professionally acceptable rather than reflecting their true attitudes, potentially underreporting stigmatizing beliefs or fears. The self-administered online format may have partially mitigated this bias compared to face-to-face interviews, but it cannot be eliminated entirely.

To minimize information bias, a questionnaire previously used in similar contexts was adapted and piloted with 20 healthcare professionals to ensure clarity and cultural appropriateness. Anonymous and confidential data collection was implemented to encourage honest responses. However, these measures cannot fully eliminate the biases described above, and findings should be interpreted with these limitations in mind.

### 2.7. Study Size

The sample size was calculated using the formula for an infinite population proportion (>10,000), using a 95% confidence level (Z = 1.96) and a 5% margin of error (ε = 0.05), leaving the probability of the event to chance (*p* = 0.5).

Following the formula = Z^2^⋅*p* × (1 − *p*)/ε^2^; n = (1.96)^2^ × 0.5⋅(1 − 0.5)/(0.05)^2^ ≈ 384.

Therefore, it was determined that at least 384 participants were needed for the sample to be representative. Ultimately, 1013 healthcare professionals participated in the quantitative phase, providing robust statistical power for descriptive and comparative analyses within the sample. In the qualitative phase, purposive sampling was used, contacting 12 professionals interested in participating, all of whom accepted.

### 2.8. Quantitative Variables

Quantitative variables were mainly discrete, including age, years of professional experience, and ratings of fear of HIV infection. The Kolmogorov–Smirnov normality test was applied to determine whether their distribution was symmetrical or asymmetrical.

### 2.9. Statistical Methods

Quantitative data were analyzed using IBM SPSS Statistics Version 29 (IBM Corp., Armonk, NY, USA). Descriptive statistics included absolute and relative frequencies for categorical variables and medians with interquartile ranges (IQRs) for continuous variables. The Kolmogorov–Smirnov test was applied to continuous variables (age, years of experience, fear ratings) to assess normality; all showed significant departures from normality (*p* < 0.05), justifying the use of non-parametric tests.

For group comparisons, the Kruskal–Wallis test was used to assess differences in ordinal responses (e.g., fear of contagion, preparedness) across gender and professional categories. When significant differences were detected, post hoc pairwise comparisons were conducted using the Dunn-Bonferroni method to control for multiple comparisons. Effect sizes were calculated using epsilon-squared (ε^2^), with values of 0.01, 0.06, and 0.14 interpreted as small, medium, and large effects, respectively.

For categorical variables, chi-square tests of independence were used. When expected cell counts were below 5 in more than 20% of cells (particularly for the non-binary group, n = 26), Fisher’s exact test or Monte Carlo simulation (10,000 samples) was applied as appropriate. Cramer’s V was reported as a measure of effect size for chi-square tests.

The Friedman test was used to compare related ordinal variables (multiple fear-related items within the same participants). Significant Friedman tests were followed by Durbin-Conover post hoc pairwise comparisons with Bonferroni correction.

Given the exploratory nature of the study and the large number of comparisons, a Bonferroni correction was applied to primary comparisons (α = 0.05/number of primary comparisons). For secondary exploratory analyses, uncorrected *p*-values are reported alongside effect sizes, with interpretation emphasizing clinical and practical significance rather than statistical significance alone. Confidence intervals (95% CI) are reported for key proportions where appropriate.

For the non-binary group (n = 26), statistical power was limited, and results should be interpreted with caution. Analyses involving this group are presented for completeness but are considered exploratory.

### 2.10. Qualitative Analysis

Qualitative data were analyzed using reflexive thematic analysis. Analysis was primarily inductive, allowing themes to emerge from the data, with some deductive elements guided by the study’s conceptual framework (knowledge, attitudes, stigma).

Two researchers (YAPJ and IDLM) independently coded the first three transcripts to develop an initial coding framework. Initial independent coding yielded 47 codes from the first author and 52 codes from the second author, with approximately 78% overlap in code content. Discrepancies were discussed in three dedicated meetings, during which codes were compared, definitions clarified, and disagreements resolved through discussion until consensus was reached. A consensus codebook containing 38 final codes organized into 12 subcategories was created. The remaining transcripts were coded by the first author, with bi-weekly meetings with the second author to discuss emerging themes, review coding decisions, and ensure consistency. During these meetings, approximately 15% of coded segments were reviewed by the second author to verify coding accuracy; no substantial disagreements emerged. ATLAS.ti Version 24 (ATLAS.ti Scientific Software Development GmbH, Berlin, Germany) software was used to manage coding and facilitate thematic organization.

Codes were grouped into preliminary themes, which were reviewed against the dataset to ensure they accurately represented the data. Themes were refined through iterative discussion among the research team. An audit trail documenting coding decisions, theme development, and analytical memos was maintained throughout the process.

Credibility was enhanced through member checking, peer debriefing, and inclusion of diverse participant perspectives. Dependability was supported by maintaining detailed records of analytical decisions. Transferability is supported by a thick description of the context and participant characteristics.

### 2.11. Ethical Considerations

The study followed the principles of the Declaration of Helsinki and the European Regulation 2016/679 on Data Protection. Participants provided informed consent, which explicitly included permission for the publication of anonymized responses and direct quotes. The study was approved by the Clinical Research Ethics Committee of Bellvitge Hospital in Cataluña (Act 03/24, Ref. PR352/23).

## 3. Results

### 3.1. Quantitative Phase

A total of 1021 responses were obtained, of which eight forms were discarded due to incomplete data, leaving 1013 valid responses. Based on social media analytics, it was estimated that the survey invitation reached approximately 3000 healthcare professionals. However, given the nature of social media dissemination, this figure represents potential reach rather than a defined eligible population, and therefore a conventional response rate cannot be accurately calculated. Approximately 34% of the estimated reach resulted in completed surveys, though this should be interpreted as a participation estimate rather than a true response rate.

#### 3.1.1. Characteristics of the Surveyed Population

Most respondents were female (57.6%), followed by male (39.9%) and non-binary (2.6%) participants. The median age of participants was 38 years (interquartile range, IQR = 13), with only minor variations between genders. Technicians constituted the largest professional group (30.3%), followed by nurses (28.5%) and physicians (22.8%). The majority of participants were employed within the public health system (65.5%), with outpatient care representing the most common area of practice (31.7%) (see Table 1).

**Figure 1:**
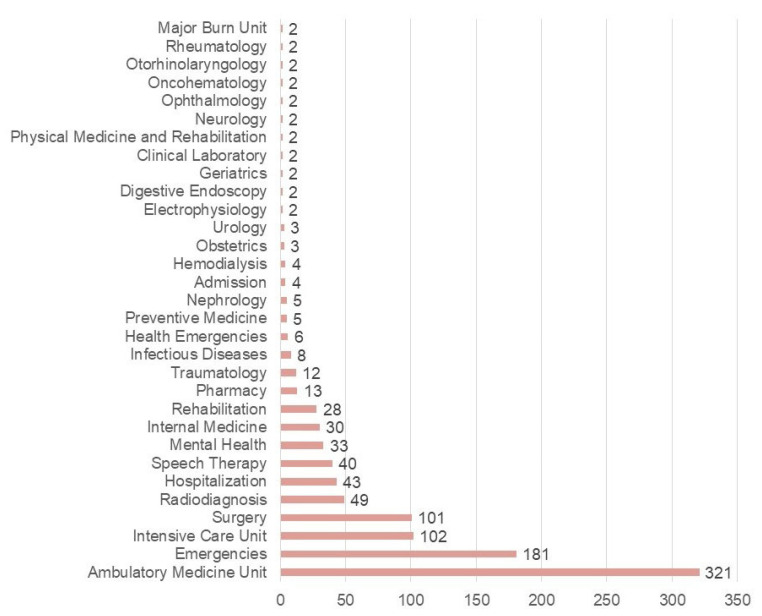
Distribution of participants according to the service in which they work (N = 1013).

**Figure 2:**
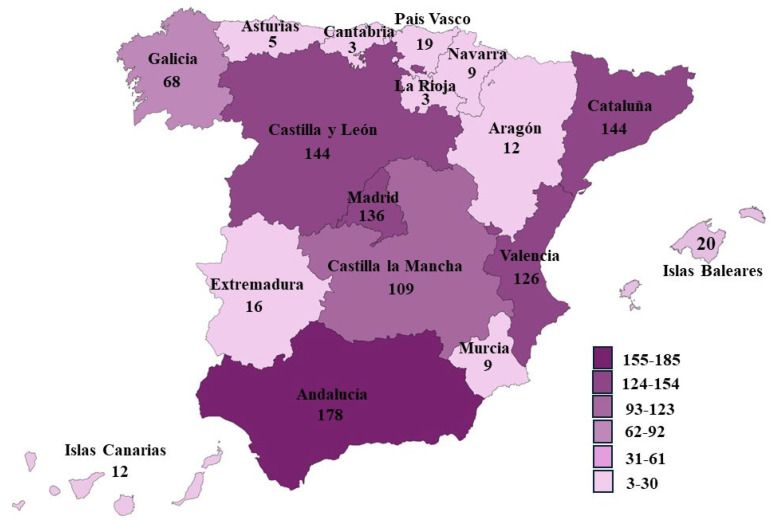
Distribution of participants according to the autonomous community of origin (N = 1013). Values represent the number of participants.

Median professional experience was 12 years (IQR = 13.3), with slight differences observed across gender and professional categories (Figure 1). Geographically, Andalusia accounted for the highest proportion of participants (17.6%), followed by Catalonia and Castilla y León (14.2% each). The distribution of respondents across autonomous communities is illustrated in Figure 2.

#### 3.1.2. Training and Previous Experiences with People Living with HIV

Participants were asked if they had met PLWH outside the workplace; 50.4% (n = 511) said they had, and 49.6% (n = 502) said they had not. Participants were also asked about their participation in training related to counselling, voluntary HIV testing, or care for PLWH; 54.5% (n = 552) indicated that they had not received such training. Furthermore, 57.9% (n = 586) indicated they did not feel prepared to provide health care to PLWH.

#### 3.1.3. Knowledge About the Spread and Transmission of HIV

The majority of participants (90.4%, n = 916) identified unprotected oral sex as a risk condition for HIV transmission. Similarly, nearly all respondents (99.4%, n = 1007) recognized that sharing needles for administering medication, tattoos, or drug use constitutes a risk factor. Most participants (97.5%, n = 988) also considered receiving blood transfusions from individuals who tested negative for HIV to be a risk, while a substantial proportion (93.5%, n = 947) regarded sharing razors as dangerous.

Conversely, bathing in the same water as a PLWH was ruled out as a risk by 98.4% of respondents (n = 997). Likewise, 90.7% (n = 919) indicated that receiving a bite from a mosquito that had previously bitten a PLWH does not represent a risk. In addition, 92% of participants (n = 932) dismissed the possibility of HIV transmission through sharing cutlery and plates.

#### 3.1.4. Knowledge About Factors That Can Reduce the Risk of HIV Infection

Regarding factors that can reduce the risk of HIV infection, 90.9% (n = 921) of participants indicated that abstaining from sexual intercourse would reduce the risk. In contrast, 88.1% (n = 892) reported that maintaining a healthy diet would not reduce transmission risk. The vast majority considered the use of a new needle for each injection (99.6%, n = 1009) and the use of condoms during sexual intercourse (99.4%, n = 1007) as effective risk reduction measures.

#### 3.1.5. Beliefs About HIV Infection

Regarding beliefs about HIV infection, 93.6% (n = 948) of participants indicated that having more sexual partners increases the risk of acquiring HIV. Most respondents (97.4%, n = 987) stated that it is not possible to identify a PLWH solely by appearance. Furthermore, 88.8% (n = 900) recognized that a person could still be infected despite a negative HIV test result.

Concerning the application of biosecurity measures, 96.3% (n = 976) agreed that healthcare providers should apply the same measures to all patients. Regarding mother-to-child transmission of HIV, 89.4% (n = 906) affirmed that antiretroviral treatment for the mother reduces the risk. A caesarean section was considered a preventive measure by 74.4% (n = 754), and the 78.4% (n = 794) identified refraining from breastfeeding as a risk-reduction strategy. Additionally, 90.7% (n = 919) indicated that promoting prenatal check-ups during pregnancy contributes to reducing transmission risk.

#### 3.1.6. Treatment Knowledge for People Living with HIV

The majority of respondents (96.7%, n = 980) indicated that only the patient should be informed of a positive HIV diagnosis, while 3.3% (n = 33) considered that both the patient and their family should be informed. Similarly, 91.6% (n = 928) stated that the result should be communicated exclusively to the patient rather than to both the patient and their partner.

When asked about disclosure in a personal context, 77.8% (n = 788) stated they would keep a family member’s HIV status confidential from friends. In terms of professional responsibility, 68.2% (n = 691) affirmed that only the patient should be informed of a positive result, whereas 22.5% (n = 228) believed the Ministry of Health should also be notified.

Awareness of antiretroviral therapy was high, with 98.1% (n = 994) reporting familiarity with the treatment. Most participants (97.4%, n = 987) agreed that antiretroviral therapy prolongs the life expectancy of PLWH. While 90.4% (n = 916) recognized that it does not cure HIV, 94.6% (n = 958) affirmed its capacity to reduce transmission risk. Additionally, 67% (n = 679) indicated that antiretroviral therapy frequently produces side effects that cause physical discomfort in patients

#### 3.1.7. Attitudes Towards People Living with HIV

A substantial majority of participants (93.3%, n = 945) agreed that an HIV-positive woman has the right to become pregnant. Similarly, 97.7% (n = 990) supported that a teacher living with HIV should continue teaching as long as their health allows. Most respondents (83.9%, n = 850) rejected the notion that most PLWH acquire the infection through irresponsible behavior.

The willingness to provide care was also notable, with 95% (n = 970) indicating they would care for a family member with AIDS at home. Additionally, 70.3% (n = 712) believed their partner would remain with them if diagnosed with HIV. High levels of acceptance were also observed in other scenarios; for example, 90.4% (n = 916) expressed no objection to sharing a bathroom with a PLWH, and 87.7% (n = 888) reported no concern about purchasing food from a supplier living with HIV. Finally, nearly all participants (99.4%, n = 1007) strongly disagreed with the idea that HIV and AIDS are a punishment from God for immorality.

#### 3.1.8. Fear of HIV Infection

Respondents were asked if they would be nervous if they realized their next patient was a PLWH, and 90.7% (n = 919) said they would not be nervous. When asked about their concern about being turned away from seeing PLWH, 93.3% (n = 945) said they would not be nervous. However, regarding their concern about becoming infected with HIV, 60.3% (n = 611) indicated that this was a situation that worried them.

Regarding the possibility that some patients would stop coming to their office when they found out they were treating PLWH, 90.1% (n = 913) said they would not be worried. Regarding whether other people would think that he or she is also living with HIV, 92.6% (n = 938) said they were not worried at all. Finally, 68.9% (n = 698) expressed concern about not having received enough training on HIV, while 26.8% (n = 271) were worried about the risk of becoming infected.

More responses for each of these sections are presented in Table 2.

#### 3.1.9. Statistical Analysis

Although gender differences were not a primary objective of this study, exploratory analyses were conducted by gender because it constitutes a relevant sociodemographic characteristic of healthcare professionals that may influence knowledge, attitudes, and stigma. In addition, statistical analyses were performed by crossing independent variables (such as gender and professional category) with dependent variables (including knowledge, attitudes, training, and fear of contagion). These analyses revealed significant associations in specific areas, particularly regarding training needs and fear of contagion.

A significant disparity was observed in the distribution of professional roles by gender (χ^2^ = 98.4, *p* < 0.001, Cramer’s V = 0.22). Females were significantly overrepresented among nurses (72.3%) and nutritionists (87.5%), while males constituted the majority of dentists (75%) and technicians (52%). Non-binary individuals, albeit a small group, had a significantly higher representation among psychologists (14.7%) compared to other professions.

Regarding knowledge, while overall levels were high, a gender-based analysis of a key misconception revealed a notable difference. Significantly more males (2.2%) and females (1.7%) incorrectly believed HIV could be transmitted via mosquito bites, compared to no non-binary individuals holding this belief (χ^2^ = 8.1, *p* = 0.017). Furthermore, when analyzing the feeling of preparedness, a Kruskal–Wallis test identified significant differences based on gender (χ^2^ = 9.8, *p* = 0.007), with a small effect size (ε^2^ = 0.010), indicating that female and non-binary professionals reported feeling less prepared (“Nothing” prepared: Female 24.1%, Non-binary 50%; Male 15.7%) despite similar training participation rates.

Also, the Kruskal–Wallis test identified significant differences in concern about the HIV/AIDS training received based on gender (χ^2^ = 12.3, *p* = 0.002), with a small effect size (ε^2^ = 0.012). This indicates a higher level of concern among females.

An analysis of fear of contagion by professional category using the Kruskal–Wallis test also showed significant differences (χ^2^ = 45.2, *p* < 0.001, ε^2^ = 0.045). Post hoc comparisons using the Dunn-Bonferroni method revealed that technicians reported the highest levels of fear (“A lot”: 3.9% of technicians), which was significantly greater than the fear reported by physicians (0.4%, *p* < 0.001) and nurses (1.4%, *p* = 0.005).

Significant differences were also found in healthcare professionals’ perceptions of the likelihood that their patients would stop attending the clinic upon learning that they treated PLWH (χ^2^ = 25.8, *p* = 0.002). The effect size was moderate (ε^2^ = 0.025), with the most notable differences observed between physicians and other professionals (*p* = 0.003) and between nurses and other professionals (*p* = 0.007).

Regarding fear of contagion, the results showed significant differences in moderate magnitude (χ^2^ = 52.6, *p* < 0.001, ε^2^ = 0.052). The most prominent differences were between physicians and technical professionals, and between nurses and technical professionals (*p* < 0.001 for both).

Finally, the Friedman test applied to all fear of contagion variables was significant (χ^2^ = 2674, *p* < 0.001). Post hoc Durbin-Conover comparisons with Bonferroni correction indicated an association between concern about HIV/AIDS training and fear of contagion (*p* < 0.001), suggesting that sufficient training is related to a lower level of concern. Similarly, fear of contagion was lower among professionals who were less worried about being rejected for caring for PLWH (*p* < 0.001).

### 3.2. Qualitative Phase

**Figure 3:**
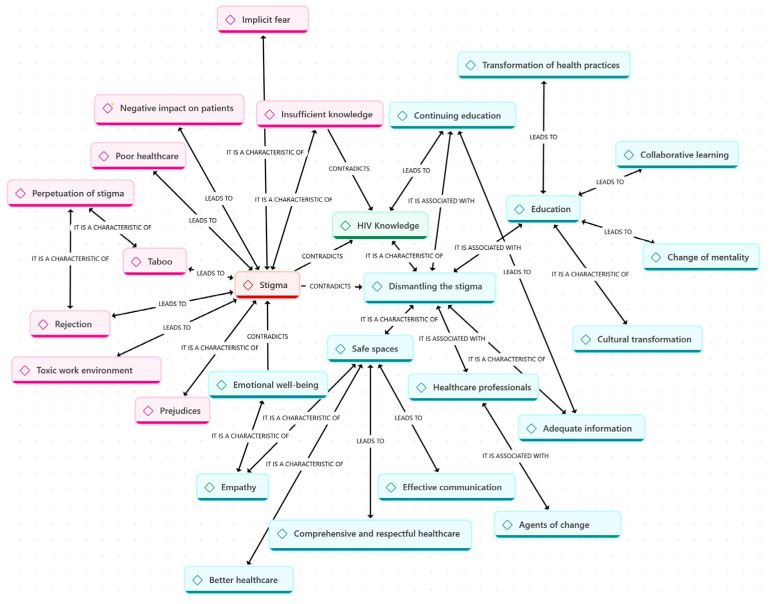
Code map of the qualitative analysis showing relationships between major themes (n = 12 participants). Themes include: HIV training and knowledge, experiences with stigma in the workplace, barriers to stigma-free care, and strategies to reduce stigma. Lines indicate thematic connections identified during analysis.

Twelve professionals were interviewed, including three nursing assistants, two nurses, three doctors, two psychologists, one physiotherapist, and one nutritionist. The code map of the qualitative analysis is shown in Figure 3.

#### 3.2.1. HIV Training and Knowledge

Several participants indicated that they had not taken specific courses on HIV, aligning with the concern expressed in the quantitative phase, where nearly 60% reported insufficient training.

*“Female nursing assistant, 29 years old, Andalucía”: “I have not had any specific training, only the basics of my degree, and that is not enough to properly treat these patients.”*

Participants described how the lack of training had a direct impact on their professional practice. Some explained that inadequate knowledge created uncertainty and even risked stigmatizing patients. This highlighted the importance of ongoing training and learning in the profession to ensure patients’ respectful and sensitive treatment.

*“Female nutritionist, 40 years old, Madrid”: “My knowledge impacts how I treat my patients. If I do not know enough, I can make them feel uncomfortable or stigmatized.”*

#### 3.2.2. Experiences with Stigma in the Workplace

Participants described stigma in diverse ways, often framing it as a judgment that altered professional–patient interactions:

*“Male nurse, 35 years old, Valencia“: “It is a prior judgment that affects the care we provide. It is as if, knowing that someone has HIV, we already look at them differently.”*

Also, some participants agreed that stigma towards people living with HIV persists due to a lack of information and cultural prejudices.

*“Female psychologist, 45 years old, País Vasco”: “The stigma comes from ignorance. People still associate HIV with immoral behavior.”*

As for their willingness to interact with people living with HIV, several participants were open. However, some admitted to having fears, especially about the judgements others might make about their condition.

*“Male doctor, 50 years old, Andalucía,”: “I feel good about caring for these people, but sometimes I fear that others will judge me for it.”*

Others recalled situations in which they had witnessed discriminatory behavior in their workplace, such as colleagues avoiding patient contact:

*“Male nursing assistant, 26 years old, Cataluña”: “I have seen colleagues avoid patients with HIV. It is not correct, but it happens. For example, they do not take their vital signs to avoid touching them.”*

Despite the situations mentioned above, the professionals agreed that above the stigma lies the right to be treated with dignity and to health care, especially in a country like Spain, where public health is advocated; in this regard, one of the professionals commented:

*“Male physiotherapist, 56 years old, Castilla la Mancha”: “Patients should not be afraid. Care is a right, and professionals are willing to help without judging.”*

However, not all participants shared this optimistic view. One professional offered a more critical perspective, acknowledging that the gap between professional ideals and actual practice remains significant:

*“Female doctor, 36 years old, Extremadura”: “We like to think we treat everyone equally, but the reality is different. I have seen how some colleagues, even unconsciously, change their behavior when they know a patient has HIV. They may not refuse care, but there are subtle differences—less time spent, more distance, excessive use of protective equipment. We are not as stigma-free as we would like to believe.”*

This contrasting perspective highlights that while overt discrimination may be rare, subtle forms of differential treatment persist, underscoring the need for interventions that address not only explicit attitudes but also implicit biases and unconscious behaviors.

#### 3.2.3. Barriers to Providing Stigma-Free Care

Participants repeatedly identified fear of contagion and lack of training as barriers to providing care without stigma.

*“Female psychologist, 45 years old, País Vasco”: “Lack of training and misinformation are the biggest barriers. If professionals are not trained, they cannot provide adequate care. Lack of knowledge is as dangerous as the stigma itself.”*

One of the nurses also commented:

*“Female nurse, 30 years old, Aragón”: “Many people fear that, by treating a patient with HIV, they will become infected, which is completely wrong. You must take precautions, as you would with any other patient.”*

#### 3.2.4. Strategies to Reduce Stigma

Professionals proposed various strategies to mitigate stigma in the healthcare setting. These proposals reflected the need for a multidimensional approach to address stigma and foster more empathetic and respectful care for those living with HIV.

*“Female doctor, 28 years old, Castilla y León”: “More awareness and training programs on HIV should be implemented for all levels of health personnel, as this can help create a more inclusive and understanding environment.“*

*“Male psychologist 33, Galicia”: “Conduct interactive workshops that encourage open dialogue and empathy towards people living with HIV, as this can demystify many of the existing prejudices.”*

Some professionals described their own attempts to introduce these strategies into their work environment, acknowledging mixed results:

*“Female nutritionist, 40 years old, Madrid”: “I have tried to talk openly about HIV with my colleagues, but I have found that not everyone is willing to engage in the conversation. We need to create safe spaces where they feel comfortable expressing their doubts and concerns.“*

Others highlighted the role of institutions, stressing the need for campaigns that humanize the experience of living with HIV:

*“Female doctor, 36 years old, Extremadura”: “Institutions must promote awareness campaigns that include testimonies from people living with HIV to humanize the experience and help break down stigmas.“*

Continuing the dialogue, some professionals emphasized that addressing stigma requires moving beyond isolated initiatives and calls for structural changes within healthcare institutions:

*“Male doctor, 50 years old, Andalucía”: “It is not enough to offer sporadic courses; stigma is a cultural and institutional problem. We need continuous training integrated into our work routines, using real cases and testimonies. If we do not normalize this conversation in hospitals and primary care, stigma will remain hidden.“*

From a mental health perspective, psychologists highlighted the emotional dimension of stigma, which affects both patients and professionals:

*“Male psychologist, 33 years old, Galicia”: “Many professionals fear being judged by their colleagues or society for working closely with patients living with HIV. This fear is seldom addressed, and it is crucial to create spaces where healthcare workers can express and reflect on their biases. Without such self-awareness, no training will be truly effective.“*

Finally, nurses who are often on the front lines of patient care stressed that combating stigma requires practical changes in daily interactions, not just theoretical knowledge.

*“Female nurse, 30 years old, Aragón”: “Stigma is dismantled through small acts: taking vital signs, touching a patient’s hand, or explaining a treatment without prejudice. Training helps, but what truly transforms care is practicing empathy daily and seeing the person beyond the disease.“*

These reflections indicate that mitigating stigma demands a multidimensional approach combining continuous training, safe spaces for professional reflection, and strong institutional commitment. The depth of these testimonies underscores that addressing stigma involves not only increasing knowledge but also transforming attitudes and organizational culture.

### 3.3. Integration of Quantitative and Qualitative Findings

Table 3 presents a joint display integrating key quantitative findings with corresponding qualitative themes, demonstrating how qualitative data explain and contextualize quantitative patterns.

This integration reveals that while healthcare professionals in Spain demonstrate strong factual knowledge and express positive attitudes toward PLWH, persistent fear and stigma are driven primarily by insufficient practical training and the gap between theoretical knowledge and clinical confidence. Qualitative data contextualize quantitative patterns, revealing that self-reported attitudes may be influenced by social desirability, and that observed stigma in colleagues suggests ongoing challenges despite overall positive trends.

## 4. Discussion

### 4.1. Main Findings

This mixed-methods study of 1013 healthcare professionals in Spain revealed high levels of factual knowledge about HIV transmission, prevention, and treatment, alongside generally positive attitudes toward the rights and dignity of PLWH. However, significant gaps persist: more than half of participants reported receiving no specific HIV training and feeling unprepared to provide care, and fear of contagion remained prevalent despite accurate knowledge. Qualitative interviews contextualized these findings, revealing that stigma persists primarily due to insufficient practical training and cultural prejudices, and that the gap between knowledge and confidence represents a critical barrier to stigma-free care.

Key quantitative findings include: (1) over 90% correctly identified major transmission routes and prevention methods; (2) 54.5% had received no HIV-specific training, and 57.9% felt unprepared to care for PLWH; (3) 60.3% expressed concern about becoming infected despite high knowledge; (4) attitudes were largely positive, with over 93% supporting reproductive rights and rejecting moral judgments; and (5) significant differences emerged by professional category, with technicians reporting higher fear and less preparedness than physicians or nurses.

Qualitative themes illuminated the mechanisms underlying these patterns: participants explicitly linked fear and stigma to training deficits, described witnessing discriminatory behaviors in colleagues (e.g., avoiding physical contact), and emphasized that knowledge alone is insufficient without practical skills and emotional preparation. Integration of quantitative and qualitative findings revealed that self-reported attitudes may be influenced by social desirability, and that observed stigma suggests ongoing challenges despite positive self-reports.

### 4.2. Comparison with Existing Literature

The demographic profile of our sample aligns with previous research. Most respondents were women and had a technical or specialized background, similar to what has been reported in other studies [14,15]. The diversity of educational backgrounds observed, despite all participants being healthcare personnel, suggests that specific HIV training may be lacking across professional categories, a finding corroborated by the fact that more than half reported not having received such training.

Regarding HIV knowledge, our findings are encouraging yet reveal important nuances. The high proportion of professionals who correctly identified transmission risks is consistent with studies showing that healthcare workers generally possess accurate factual knowledge. Similarly, attitudes toward confidentiality reflected a strong understanding of medical ethics and patient privacy, aligning with the bioethical principle of autonomy and European data protection regulations [16,17,18]. However, as previous research has demonstrated, even accurate knowledge does not guarantee stigma-free care; any level of HIV-related stigma from healthcare providers can compromise patient participation and health outcomes [19,20].

The positive attitudes observed in our study represent progress compared to earlier research. The majority of respondents supported the reproductive rights of HIV-positive women and the continued employment of HIV-positive teachers. In contrast, Spence et al. [14] found that up to 20% of participants did not agree that PLWH should be allowed to have children. This shift suggests evolving attitudes, possibly reflecting changes in HIV education and public awareness over time. Furthermore, the rejection of the notion that HIV results from “irresponsible behavior” indicates a more nuanced understanding of HIV acquisition, moving away from moralistic framings of the disease.

However, concerns about stigma and discrimination persisted, especially in the health care setting, where the quality of care was affected by fear of infection and omissions in treatment, as reported by other authors [21,22]. Professionals acknowledged that lack of information and cultural biases contributed to this stigma but also reaffirmed their ethical commitment to treating patients with dignity and respect, similar to what has been reported in other works [23,24]. Some previous studies have pointed out that when accessing health care, PLWH often experienced anticipated stigma related to lower trust in health care professionals and poor engagement with their treatment [25,26,27,28,29]. Case reports from Central and Eastern Europe highlighted the discrimination they faced, such as the testimony of a patient in Serbia who stated that “the worst stigma comes from health care workers” and other patients who report seeing care providers take more biosecurity measures to care for them [30,31,32]. In this European region, they were often denied treatment and sometimes made to wait for long periods in isolated rooms [25,30,32,33]. Refusal to treat PLWH has sometimes been falsely justified because facilities or providers were not well equipped to care for people with HIV [25,30,34]. Indeed, in the study by Spence et al. [14], low HIV knowledge was found to be associated with increased HIV prejudice and stigma (*p* = 0.0391); in the study by Vaughan et al. [15], findings highlight the critical role of knowledge in reducing stigma-related behaviors and fears among health care workers.

Additionally, anticipated stigma has been shown to deter many patients from utilizing HIV testing and care services, resulting in late presentations for necessary care [31,35,36,37]. This phenomenon not only constitutes an obstacle to accessing health services but also exacerbates the HIV pandemic, hampering efforts to control the disease [35,38]. Critical attitudes and stigma of health care workers during clinical visits create a hostile environment that discourages patients from seeking care [35,39,40]. This stigma becomes a public health problem, as it delays diagnosis and treatment, perpetuating the transmission of the virus and hindering the World Health Organization’s goals for greater disease control [35,39].

### 4.3. Plausible Explanations and Mechanisms

The disconnect between high knowledge and persistent fear can be understood through several mechanisms. First, knowledge of transmission routes is primarily cognitive, while fear of contagion is emotional and may be driven by perceived risk rather than actual risk. Second, insufficient practical training in biosafety procedures and post-exposure protocols may leave professionals feeling vulnerable despite theoretical understanding. Third, cultural and historical associations between HIV and marginalized groups may perpetuate implicit biases that coexist with explicit egalitarian attitudes, a phenomenon documented in social psychology as “implicit stigma.”

The qualitative finding that stigma is “dismantled through small acts” of empathetic care suggests that experiential learning and role modeling may be more effective than didactic knowledge transmission alone. This aligns with educational theories emphasizing the importance of reflective practice, supervised clinical experience, and normalization of interactions with PLWH in reducing stigma.

The higher fear among technicians may reflect not only training gaps but also differences in professional identity and empowerment. Physicians and nurses may have greater access to continuing education, more opportunities to ask questions and seek clarification, and stronger professional socialization emphasizing patient-centered care. Addressing stigma therefore requires attention to professional hierarchies and equitable access to training across all healthcare roles.

The possibility that professionals’ self-assessment is biased by the desire to project a positive image (social desirability bias) may hide prejudices. This is supported by qualitative accounts in which participants more readily described stigmatizing behaviors in colleagues than in themselves, suggesting that actual stigma may be higher than self-reported attitudes indicate.

### 4.4. Practical Implications

Findings suggest several actionable strategies for reducing HIV-related stigma in Spanish healthcare settings:

1. Implement standardized, mandatory training on HIV for all healthcare professionals: Training should cover not only transmission and treatment facts but also practical biosafety skills, post-exposure protocols, communication strategies, and stigma awareness. Content should be tailored to different professional roles (physicians, nurses, technicians, allied health) and include both initial and continuing education components. This addresses the finding that 54.5% had received no HIV-specific training and 57.9% felt unprepared to care for PLWH.
2. Develop interprofessional stigma-reduction workshops: Interactive workshops should incorporate testimonies from PLWH, reflective exercises on implicit bias, role-playing scenarios, and facilitated discussions in safe spaces where professionals can express doubts and fears without judgment. These workshops should emphasize empathy, humanization, and the impact of stigma on patient outcomes. This recommendation emerged directly from qualitative interviews and addresses the emotional dimension of fear that persists despite knowledge.
3. Strengthen institutional commitment and protocols: Healthcare institutions should establish clear policies normalizing HIV care, ensuring confidentiality, providing accessible post-exposure prophylaxis, and creating accountability mechanisms for discriminatory behavior. Stigma reduction should be integrated into quality improvement initiatives, with monitoring of patient-reported experiences and staff training compliance. This addresses the institutional and cultural dimensions of stigma identified in qualitative themes.

These strategies should be implemented at multiple levels—individual (knowledge and attitudes), interpersonal (communication and empathy), and institutional (policies and culture) to achieve sustainable stigma reduction. The multidimensional nature of these recommendations reflects the complexity of stigma as both an individual and structural phenomenon.

### 4.5. Strengths, Limitations, and Future Directions

This study has several strengths, including a large and geographically diverse sample (N = 1013) covering several autonomous communities of Spain, a mixed-methods design that provides both breadth and depth, and explicit integration of quantitative and qualitative findings through a joint display table. The inclusion of diverse healthcare professions and settings (public and private, hospital and primary care) increases the relevance of findings across the Spanish healthcare system. The use of an adapted and piloted instrument with acceptable internal consistency (Cronbach’s α = 0.74–0.81), combined with rigorous qualitative analysis following COREQ guidelines, strengthens methodological rigor.

However, important limitations must be acknowledged. Non-probability convenience sampling through social media introduces significant selection and coverage bias, limiting generalizability to the entire population of Spanish healthcare professionals. The estimated 34% participation rate is based on social media reach metrics rather than a defined eligible population, and the characteristics of non-responders remain unknown. Social desirability bias is a significant concern; despite anonymity, participants may have underreported stigmatizing attitudes, and qualitative accounts of observed stigma in colleagues suggest actual stigma may exceed self-reported levels. The cross-sectional design precludes causal inference regarding relationships between training, knowledge, fear, and stigma. The non-binary group (n = 26) was too small for robust statistical analysis. The instrument, while adapted and piloted, was not formally validated using rigorous psychometric methods in the Spanish context. The qualitative sample (n = 12), though achieving thematic saturation, may not capture the full diversity of experiences across all regions and specialties. Finally, findings reflect the Spanish healthcare context and may not be transferable to other countries with different healthcare systems or cultural attitudes toward HIV.

Future research should employ probability sampling methods to obtain representative estimates and allow generalization. Longitudinal and experimental designs (e.g., randomized controlled trials of training interventions) are needed to establish causality and evaluate intervention effectiveness. Qualitative research with larger samples, including PLWH perspectives on experienced stigma in healthcare settings, would provide richer contextual understanding and center patient voices. Development and validation of culturally appropriate stigma measurement tools for Spanish healthcare settings, including measures of implicit bias and observed behaviors rather than self-report alone, would enhance future research quality and enable more accurate assessment of stigma prevalence and change over time.

## 5. Conclusions

Healthcare professionals in Spain demonstrate high levels of factual knowledge about HIV transmission, prevention, and treatment, and express generally positive attitudes toward the rights and dignity of PLWH. However, significant gaps persist in practical training and clinical preparedness, and fear of contagion remains prevalent despite accurate knowledge. Qualitative findings reveal that stigma persists due to insufficient training, cultural prejudices, and the gap between theoretical knowledge and emotional confidence. These findings underscore that knowledge alone is insufficient to eliminate HIV-related stigma; structured, continuous, and experiential training is essential to translate knowledge into stigma-free practice.

Based on these findings, we propose the following evidence-informed recommendations:

1. Implement standardized, mandatory training on HIV for all healthcare professionals: Training should cover not only transmission and treatment facts but also practical biosafety skills, post-exposure protocols, communication strategies, and stigma awareness. Content should be tailored to different professional roles (physicians, nurses, technicians, allied health) and include both initial and continuing education components.
2. Develop interprofessional stigma-reduction workshops: Interactive workshops should incorporate testimonies from PLWH, reflective exercises on implicit bias, role-playing scenarios, and facilitated discussions in safe spaces where professionals can express doubts and fears without judgment. These workshops should emphasize empathy, humanization, and the impact of stigma on patient outcomes.
3. Strengthen institutional commitment and protocols: Healthcare institutions should establish clear policies normalizing HIV care, ensuring confidentiality, providing accessible post-exposure prophylaxis, and creating accountability mechanisms for discriminatory behavior. Stigma reduction should be integrated into quality improvement initiatives, with monitoring of patient-reported experiences and staff training compliance.

Addressing HIV-related stigma in healthcare settings requires a multidimensional approach combining individual education, interprofessional training, and institutional culture change. Only through sustained commitment at all levels can the Spanish healthcare system achieve truly equitable, respectful, and stigma-free care for people living with HIV.

## Figures and Tables

**Table 1:** Characteristics of participants, broken down by gender.

	**Female**		**Male**		**Non-Binary**		**Total**
**Age**	**Med**	**IQR**	**Med**	**IQR**	**Med**	**IQR**	
In years	37	13.0	39	14.0	41	12.8	
**Education Level**	**n**	**%**	**n**	**%**	**n**	**%**	**n**
Doctorate	19	48.7%	20	51.3%	0	0.0%	39
Clinical specialty via the Ministry of Health for doctors, nurses, psychologists, pharmacists, etc.	200	67.3%	92	31.0%	5	1.7%	297
Other clinical specialties	3	60.0%	2	40.0%	0	0.0%	5
Bachelor’s degree	82	69.5%	35	29.7%	1	0.8%	118
Master’s degree	129	61.1%	79	37.4%	3	1.4%	211
Post-graduate	19	57.6%	14	42.4%	0	0.0%	33
Post-doctorate	2	66.7%	1	33.3%	0	0.0%	3
Technician	129	42.0%	161	52.4%	17	5.5%	307
***Total***	583	57.6%	404	39.9%	26	2.6%	1013
**Profession**	**n**	**%**	**n**	**%**	**n**	**%**	**n**
Nurse	209	72.3%	78	27.0%	2	0.7%	289
Pharmacist	5	55.6%	4	44.4%	0	0.0%	9
Physiotherapist	13	43.3%	17	56.7%	0	0.0%	30
Speech therapist	28	70.0%	12	30.0%	0	0.0%	40
Doctor	137	59.3%	93	40.3%	1	0.4%	231
Nutritionist	21	87.5%	3	12.5%	0	0.0%	24
Dentist	7	21.9%	24	75.0%	1	3.1%	32
Other health professional	14	77.8%	4	22.2%	0	0.0%	18
Psychologist	18	52.9%	11	32.4%	5	14.7%	34
Technician (imaging, nursing, dental, emergencies)	131	42.8%	158	51.6%	17	5.6%	306
***Total***	583	57.6%	404	39.9%	26	2.6%	1013
**Health system in which you work**	**n**	**%**	**n**	**%**	**n**	**%**	**n**
Public	400	60.2%	241	36.3%	23	3.5%	664
Private	163	51.4%	151	47.6%	3	0.9%	317
Mixed	20	62.5%	12	37.5%	0	0.0%	32
***Total***	583	57.6%	404	39.9%	26	2.6%	1013
**Type of health center where you work**	**n**	**%**	**n**	**%**	**n**	**%**	**n**
Public primary care center	72	59.0%	47	38.5%	3	2.5%	122
Private medical center	117	48.8%	120	50.0%	3	1.3%	240
Private hospital	38	55.1%	31	44.9%	0	0.0%	69
Public hospital	338	61.2%	194	35.2%	20	3.6	552
Another type of health center	18	60.0%	12	40.0%	0	0.0%	30
***Total***	583	57.6%	404	39.9%	26	2.6%	1013
**Professional experience**	**Med**	**IQR**	**Med**	**IQR**	**Med**	**IQR**	
In years	11	12.8	13	14.0	17	16.3	

Abbreviations: Med: Median; IQR: The interquartile range.

**Table 2:** Participant responses discriminated by gender.

Questions	Female		Male		Non-Binary		Total
	n	%	n	%	n	%	n
*Outside of work, have you met anyone living with HIV?*							
Yes	253	25.0%	251	24.8%	7	0.7%	511
No	330	32.6%	153	15.1%	19	1.9%	502
*Have you participated in any training on counselling, voluntary HIV testing and care for patients living with HIV?*							
Yes	247	24.4%	207	20.4%	7	0.7%	461
No	336	33.2%	197	19.4%	19	1.9%	552
*With the training you have received, how prepared do you feel to provide health care to people living with HIV?*							
Nothing	244	24.1%	159	15.7%	13	1.3%	416
A little	98	9.7%	67	6.6%	5	0.5%	170
A lot	241	23.8%	178	17.6%	8	0.8%	427
*Can a person become infected with HIV by having unprotected oral sex?*							
Yes	521	51.4%	371	36.6%	24	2.4%	916
No	48	4.7%	28	2.8%	1	0.1%	77
Not sure	14	1.4%	5	0.5%	1	0.1%	20
*Can a person become infected with HIV by bathing in the same water that a PLWH bathed in?*							
Yes	4	0.4%	0	0.0%	0	0.0%	4
No	570	56.3%	401	39.6%	26	2.6%	997
Not sure	9	0.9%	3	0.3%	0	0.0%	12
*Can a person become infected with HIV by sharing needles for applying medicine, tattoos or using drugs?*							
Yes	577	57.0%	404	39.9%	26	2.6%	1007
No	6	0.6%	0	0.0%	0	0.0%	6
*Can a person become infected with HIV by receiving a blood transfusion that has not been tested negative for HIV?*							
Yes	568	56.1%	396	39.1%	24	2.4%	988
No	11	1.1%	6	0.6%	2	0.2%	19
Not sure	4	0.4%	2	0.2%	0	0.0%	6
*Can a person become infected with HIV by being bitten by a mosquito that previously bit a person living with HIV?*							
Yes	17	1.7%	22	2.2%	0	0.0%	39
No	522	51.5%	371	36.6%	26	2.6%	919
Not sure	44	4.3%	11	1.1%	0	0.0%	55
*Can a person become infected with HIV by sharing cutlery and plates with a person living with HIV?*							
Yes	25	2.5%	20	2.0%	0	0.0%	45
No	535	52.8%	371	36.6%	26	2.6%	932
Not sure	23	2.3%	13	1.3%	0	0.0%	36
*Can a person reduce the risk of becoming infected with HIV by abstaining from sexual intercourse?*							
Yes	513	50.6%	383	37.8%	25	2.5%	921
No	66	6.5%	21	2.1%	1	0.1%	88
Not sure	4	0.4%	0	0.0%	0	0.0%	4
*Could you identify a person living with HIV just by looking at it?*							
Yes	1	0.1%	2	0.2%	1	0.1%	4
No	567	56.0%	395	39.0%	25	2.5%	987
Not sure	15	1.5%	7	0.7%	0	0.0%	22
*Should a healthcare provider apply the same biosecurity measures to all patients, regardless of whether or not they are living with HIV?*							
Yes	561	55.4%	389	38.4%	26	2.6%	976
No	18	1.8%	10	1.0%	0	0.0%	28
Not sure	4	0.4%	5	0.5%	0	0.0%	9
*In your opinion, if a patient tests positive for HIV, should the clinic/hospital inform the patient and his/her family or only the patient?*							
To the patient and his/her family	17	1.7%	14	1.4%	2	0.2%	33
Only to the patient	566	55.9%	390	38.5%	24	2.4%	980
*Are HIV and AIDS a punishment from God for immorality?*							
Disagree	579	57.2%	402	39.7%	26	2.6%	1007
Neither disagree nor agree	1	0.1%	1	0.1%	0	0.0%	2
Agree	3	0.3%	1	0.1%	0	0.0%	4
*Have you ever heard about antiretroviral therapy (ART)?*							
Yes	570	56.3%	399	39.4%	25	2.5%	994
No	13	1.3%	5	0.5%	1	0.1%	19
*Does antiretroviral therapy help prolong the lives of patients living with HIV?*							
Yes	564	55.7%	398	39.3%	25	2.5%	987
No	1	0.1%	0	0.0%	0	0.0%	1
Not sure	18	1.8%	6	0.6%	1	0.1%	25
*If you were caring for patients living with HIV as part of your job, how worried would you be about being rejected by other people for caring for people living with HIV?*							
Not at all	544	53.7%	375	37.0%	26	2.6%	945
A little	27	2.7%	21	2.1%	0	0.0%	48
A little more than usual	10	1.0%	6	0.6%	0	0.0%	16
A lot	2	0.2%	2	0.2%	0	0.0%	4
*If you were caring for patients living with HIV as part of your job, how worried would you be about becoming infected with HIV?*							
Not at all	202	19.9%	111	11.0%	5	0.5%	318
A little	325	32.1%	266	26.3%	20	2.0%	611
A little more than usual	34	3.4%	23	2.3%	1	0.1%	58
A lot	22	2.2%	4	0.4%	0	0.0%	26
*If you were caring for patients living with HIV as part of your job, how concerned would you be about whether or not you have received enough training about HIV/AIDS?*							
Not at all	49	4.8%	49	4.8%	1	0.1%	99
A little	408	40.3%	298	29.4%	21	2.1%	727
A little more than usual	65	6.4%	40	3.9%	3	0.3%	108
A lot	61	6.0%	17	1.7%	1	0.1%	79

**Table 3:** Joint display: Integration of quantitative and qualitative findings.

Quantitative Finding	Qualitative Theme	Integration/Interpretation	References
**High knowledge of transmission routes** (>90% correct on most items) but **60.3% worried about becoming infected.**	**“Fear persists despite knowledge”**—Participants described how theoretical knowledge does not eliminate emotional fear, especially when training is perceived as insufficient.	Knowledge alone is insufficient to eliminate fear; the gap between cognitive understanding and emotional response suggests that training must address not only facts but also risk perception, biosafety confidence, and emotional preparedness.	Quantitative: Section 3.1.3 and Section 3.1.8; Qualitative: Section 3.2.1 and Section 3.2.3
**54.5% had no HIV-specific training; 57.9% felt unprepared** to care for PLWH	**“Lack of training as a barrier”**—Participants explicitly identified insufficient training as the primary obstacle to providing stigma-free care and linked it directly to fear and uncertainty.	The quantitative training gap is experienced qualitatively as professional unpreparedness, which participants directly connect to stigmatizing behaviors and fear. This highlights training as a critical intervention point.	Quantitative: Section 3.1.2; Qualitative: Section 3.2.1 and Section 3.2.3
**90.7% would not be nervous** seeing a PLWH patient, yet 68.9% worried about insufficient training	**“Professionalism vs. internal doubt”**—Participants described maintaining professional composure while experiencing internal uncertainty and fear of making mistakes due to inadequate preparation	This apparent contradiction reflects social desirability in self-reported nervousness and genuine concern about competence. Professionals may suppress visible nervousness but experience internal anxiety related to preparedness	Quantitative: Section 3.1.8; Qualitative: Section 3.2.1 and Section 3.2.3
**83.9% rejected the idea** that PLWH acquire HIV through irresponsible behavior; **93.3% supported reproductive rights** of HIV+ women	**“Rejection of moral judgment”**—Participants explicitly rejected moralistic framings of HIV and emphasized patients’ rights and dignity	Quantitative attitudes align with qualitative values of non-judgment and respect for autonomy, suggesting genuine progress in reducing overt moral stigma among Spanish healthcare professionals	Quantitative: Section 3.1.7; Qualitative: Section 3.2.2
Witnessed **discriminatory behaviors** reported qualitatively (e.g., colleagues avoiding contact) despite high quantitative acceptance	**“Observed vs. self-reported stigma**”—Participants more readily described stigmatizing behaviors in colleagues than in themselves, suggesting social desirability bias in self-report	Qualitative accounts of observed stigma provide critical context for interpreting quantitative self-reports, suggesting that actual stigma may be higher than self-reported attitudes indicate	Quantitative: Section 3.1.7; Qualitative: Section 3.2.2
**Technicians reported highest fear** of contagion (χ^2^ = 45.2, *p* = 0.001) compared to physicians and nurses	**“Professional hierarchy and training access”**—Technicians described having less access to specialized training and feeling less empowered to ask questions or seek clarification	Quantitative differences by profession are explained by differential access to training and professional socialization, highlighting the need for training tailored to all professional levels	Quantitative: Section 3.1.9; Qualitative: Section 3.2.1 and Section 3.2.3
**Confidentiality highly valued** (96.7% said only patient should be informed of diagnosis)	**“Ethical commitment to privacy**”—Participants emphasized confidentiality as a core professional value and expressed concern about breaches harming patient trust	Strong quantitative support for confidentiality is reinforced by qualitative emphasis on privacy as foundational to ethical care and the therapeutic relationship	Quantitative: Section 3.1.6; Qualitative: Section 3.2.2

## Data Availability

The data presented in this study are available on reasonable request from the corresponding author. The data are not publicly available due to ethical restrictions and the need to protect the confidentiality and anonymity of the study participants.

## Abbreviations

The following abbreviations are used in this manuscript:

AIDS	Acquired Immune Deficiency Syndrome
COREQ	Consolidated Criteria for Reporting Qualitative Research
HIV	Human Immunodeficiency Virus
IQR	Interquartile Range
PLWH	People Living with HIV
SPSS	Statistical Package for the Social Sciences
STROBE	Strengthening the Reporting of Observational Studies in Epidemiology
